# Serum chemokine levels predict flares of disease activity in two independent systemic lupus erythematosus cohorts

**DOI:** 10.1186/ar3944

**Published:** 2012-09-27

**Authors:** H Bilgic, T Koeuth, J Wilson, M Petri, E Baechler Gillespie

**Affiliations:** 1 University of Minnesota, Minneapolis, MN, USA; 2 Johns Hopkins University, Baltimore, MD, USA

## Background

Gene expression profiling of blood samples from SLE patients has revealed an interferon signature defined by increased expression of type I interferon (IFN)-inducible genes. In lupus patients with quiescent disease, we previously showed that elevated serum levels of IFN-inducible chemokines IP-10, MCP-1, and MIP-3b identified a subgroup of patients who were more likely to flare within the following year. The goal of this study was to derive flare risk definitions from the discovery cohort and test those definitions in an independent patient group.

## Methods

Consenting SLE patients were enrolled in the Autoimmune Biomarkers Collaborative Network (ABCoN) study from the Hopkins Lupus Cohort. Sera were isolated from blood collected in serum-separator tubes. SearchLight multiplexed immunoassays (Aushon Biosystems) were used to quantitate serum levels of IP-10, I-TAC, MCP-1, and MIP-3b. ABCoN Cohort 1 was used for discovery of significant variables for defining flare risk and nonrisk, and those risk definitions were tested in the independent Cohort 2. In 254 patients from Cohort 1 with inactive or mild disease (SLEDAI ≤4) at baseline, we tested serum chemokine levels, the IFN gene score, and clinical laboratory values. Risk definitions were assessed by Kaplan-Meier survival analysis and multivariate Cox regression. Statistically significant markers derived from Cohort 1 were tested in Cohort 2, in which 262 patients had SLEDAI ≤4 at their first study visit.

## Results

In both cohorts, patients with high chemokine scores (calculated from IP-10, MCP-1, and MIP-3b levels) had an increased frequency of future flare (Cohort 1, Kaplan-Meier *P *= 0.0001; Cohort 2, *P *= 0.003). In Cohort 1, individual markers IP-10, I-TAC, MIP-3b, and the IFN gene score identified patients who were more likely to flare (Kaplan-Meier *P *< 0.05). Risk definitions based on IP-10, I-TAC, and the IFN gene score were also significant in Cohort 2. In multivariate regression analysis, IP-10 and I-TAC were identified as significant predictors of flare (Cohort 1, *P *= 0.003; Cohort 2, *P *= 0.04).

## Conclusion

Measurement of chemokine levels in frozen samples, collected from two longitudinally followed cohorts of SLE patients, identified several chemokines that were associated with development of flare in the subsequent 12-month period of time. A combination of chemokine levels may be used to predict flare risk in SLE and to aid in patient management.

## Competing interests

EBC is entitled to receive royalties under a licensing agreement between LabCorp and the University of Minnesota.

